# Body Mass Index and Mortality in Japan: The Miyagi Cohort Study

**DOI:** 10.2188/jea.14.S33

**Published:** 2005-03-18

**Authors:** Shinichi Kuriyama, Kaori Ohmori, Chihaya Miura, Yoko Suzuki, Naoki Nakaya, Kazuki Fujita, Yuki Sato, Yoshitaka Tsubono, Ichiro Tsuji, Akira Fukao, Shigeru Hisamichi

**Affiliations:** 1 Division of Epidemiology, Department of Public Health and Forensic Medicine, Tohoku University Graduate School of Medicine.; 2 Department of Public Health, Yamagata University School of Medicine.

**Keywords:** body mass index, cohort studies, Japan, mortality

## Abstract

BACKGROUND: The relation between body mass index (BMI) and mortality is not well established. The objective of this study was to examine the association in Japanese adults.

METHODS: In 1990, 18,740 men and 20,870 women in Miyagi Prefecture in rural northern Japan (40-64 years of age) completed a self-administered questionnaire including height and weight. Cox regression was used to estimate relative risk (RR) of mortality according to levels of BMI, with adjustment for age, marital status, smoking, drinking, walking, and weight change since 20 years of age.

RESULTS: During 11 years of follow-up, 1,121 men and 567 women had died. Compared with the referent BMI category (23.0-24.9), women in the highest BMI category (BMI>30.0) had a RR of death of 1.64 (95% confidence interval (CI), 1.09-2.49) and men and women in the lowest BMI categories (BMI<18.5) had a RR of death of 2.06 (95% CI, 1.49-2.84) and 1.83 (95% CI, 1.17-2.88), respectively, after adjustment for potential confounders and after exclusion of deaths occurring in the first three years of follow-up. We did not observe significant differences in mortality for subjects with wide range of BMI (18.5 or higher in men and 18.5 to 29.9 in women).

CONCLUSIONS: The risk of death from all causes increases in lean men and women, and obese women in this cohort.

The relation between body weight and mortality remains controversial. Although the adverse health effects of obesity are widely accepted,^1^^-^^3^ the impact of obesity upon mortality is less well established and debates about the effects of leanness still continue.^4^^-^^7^ Epidemiologic studies have reported six major patterns regarding the association between body mass index (BMI), weight (kg) divided by the square of height (m), and mortality — linearly positive,^8^^-^^14^ J-shaped,^11^^-^^13^^,^^15^ U-shaped,^4^^,^^5^^,^^10^^,^^12^^,^^16^^-^^24^ inverse J-shaped,^10^^,^^19^^,^^21^^,^^24^^-^^28^ linearly inverse,^18^^,^^25^^,^^29^ or no association.^4^^,^^27^

This discrepancy have been attributed to methodological problems including failure to control for confounding by smoking and failure to exclusion of individuals with weight loss-related illness.^6^

To further examine the relation between BMI and all-cause mortality, we conducted a population-based, prospective cohort study among middle-aged Japanese men and women, with careful consideration of potential sources of biases.

## METHODS

### Study Cohort

We have reported the design of this prospective cohort study in detail elsewhere.^30^ Briefly, from June through August 1990, we delivered a self-administered questionnaire on various health habits to 51,921 subjects (25,279 men and 26,642 women) who were 40-64 years of age and lived in 14 municipalities of Miyagi Prefecture in northern Japan. The questionnaires were delivered to and collected from the subjects’ residences by members of health promotion committees appointed by the municipal governments. Usable questionnaires were returned from 47,605 subjects (22,836 men and 24,769 women), yielding a response rate of 91.7%.

The study protocol was approved by the institutional review board of Tohoku University Graduate School of Medicine. We considered the return of the self-administered questionnaires signed by the subjects to imply their consent to participate in the study.

### Body Mass Index

The baseline survey included questions on self-reports of body weight and body height, and BMI was calculated as the weight divided by the square of the height (kg/m^2^). We used BMI as a measure of total adiposity.

We grouped subjects into the following categories: BMI lower than 18.5, 18.5 to 20.9, 21.0 to 22.9, 23.0 to 24.9, 25.0 to 26.9, 27.0 to 29.9, and 30.0 or higher according to the cutoff points proposed by the World Health Organization, in which BMI ranges 18.5-24.9, 25.0-29.9 and 30.0-39.9 are defined as normal, grade 1 overweight and grade 2 overweight, respectively.^31^

We evaluated the validity of self-reported body weight and body height. Among the study subjects, 7,153 individuals had their body height and weight measured during health examinations provided by local governments in 1990. The Pearson’s correlation coefficient (*r*) between self-reported and measured values was 0.97 for body weight, 0.85 for body height, and 0.91 for BMI. Thus, self-reported height and weight at this baseline questionnaire were sufficiently valid.

### Follow-up

Of 47,605 subjects who responded to the questionnaire, we excluded 5,139 subjects who indicated that they had prior histories of cancer (n=561), stroke (n=389), myocardial infarction (n=608), kidney disease (n=1,683), or liver disease (n=2,379). We also excluded 456 subjects who had prevalent cancer, which we ascertained by record linkage to the population-based cancer registry covering the study area.^32^ We further excluded subjects who had incomplete responses for body weight or body height information (n=2,400). Consequently, 39,610 subjects (18,740 men and 20,870 women) with 1,688 deaths (1,121 men and 567 women) were included in this analysis.

We followed up vital and residential status of subjects from June 1, 1990, through March 31, 2001. For this follow-up, we established the Follow-up Committee that was consisted of Miyagi Cancer Society; Community Health Division of all 14 municipalities; Department of Health and Welfare, Miyagi Prefectural Government; and Division of Epidemiology, Tohoku University Graduate School of Medicine. The Committee periodically reviewed the Residential Registration Record of each municipality. With this review, we identified the subjects who either died or emigrated during observation. For both decedents and emigrants, we recorded the date of death or emigration. For decedents, we investigated cause of death by reviewing the death certificates of the subjects at Public Health Centers of the study area. The underlying cause of death was coded according to International Classification of Diseases, the Ninth Revision (ICD-9). We discontinued follow-up of subjects who emigrated from the study municipalities because of logistical limitations.

We counted person-years of follow-up for each subject from June 1, 1990, until the date of death, the date of emigration outside the study districts, or the end of the study period (March 31, 2001), whichever occurred first. A total of 411,226 person-years accrued. There were 1,795 subjects (4.5% of the analytic cohort) who emigrated from the study municipalities and were lost to follow-up.

### Statistical Analysis

We used Cox proportional-hazards regression to estimate relative risk (RR) and 95% confidence interval (CI) of all-cause mortality according to categories of BMI and to adjust for potentially confounding variables, using the PHREG procedure on SAS^®^ version 8.2 statistical software package (SAS Inc., Cary, NC, USA). We conducted all analyses separately for men and women.

We considered the following variables as potential confounders: age in years; weight change since 20 years old (weight loss of 5 kg or more or not); education (up to 15 years of age, 16-18, or 19 years of age or older); marital status at baseline (whether or not living with spouse); cigarette smoking (never smokers, past smokers, current smokers smoking 1-19 cigarettes per day, or current smokers smoking at least 20 cigarettes per day); alcohol drinking (never drinkers, past drinkers, current drinkers); time spent walking (less than 1 hour per day, or 1 hour or longer per day); consumption frequencies of green vegetables and oranges (almost daily, 3-4 times per week, 1-2 times per week, or 1-2 times per month or less often). Before including the above variables into the multivariate models, we conducted stratified analyses according to the categories of covariates to examine whether the association between BMI and all cause mortality was modified by these variables. Because of the statistically significant effect modification, we did not include education, consumption frequencies of green vegetables and oranges in the multivariate models presented.

We repeated all analyses after excluding the subjects who died during the first three years of follow-up. All P values were two-tailed.

## RESULTS

### Baseline Characteristics by BMI Categories

The baseline characteristics according to the BMI categories are shown in Table 1. More than half of men and women were in the BMI range from 21.0 to 24.9, 24.8% men and 28.0% women were in the BMI range from 25.0 to 29.9, and only 1.9% men and 3.2% women were in the highest category of BMI≥30.0. The proportion of subjects who lost ≥5 kg since 20 years old were 43.8% in men and 44.2% in women in the lowest category of BMI<18.5, and that proportion decreased with increase in BMI. The proportion of current smokers decreased with increased BMI, especially in men. Obese men and women were less likely to walk one hour or longer a day. The men and women who had high BMI were less likely to consume green vegetables daily. No apparent association was observed between mean age, alcohol drinking, educational background, marital status and BMI categories.

**Table 1.** Baseline characteristics of the study subjects by body mass index categories.

	Men							Women						
	
	Body Mass Index							Body Mass Index						
		
No. of Subjects	<18.5	18.5-20.9	21.0-22.9	23.0-24.9	25.0-26.9	27.0-29.9	30≤	<18.5	18.5-20.9	21.0-22.9	23.0-24.9	25.0-26.9	27.0-29.9	30≤
													
	368	2,946	5,045	5,363	3,007	1,648	363	561	3,292	5,227	5,298	3,560	2,274	658
Mean age (SD)	53.1 (8.2)	51.6 (7.9)	51.5 (7.7)	51.4 (7.6)	51.3 (7.4)	51.1 (7.4)	50.8 (7.1)	51.7 (8.1)	50.6 (7.7)	51.2 (7.4)	52.2 (7.2)	53.2 (7.2)	53.3 (7.1)	53.5 (7.2)
Mean weight (kg) (SD)	49.1 (4.6)	54.5 (4.3)	59.3 (4.8)	64.4 (5.0)	70.1 (5.5)	75.6 (5.9)	82.9 (8.8)	42.3 (3.8)	47.0 (3.7)	51.3 (3.8)	55.8 (3.9)	59.7 (4.2)	64.9 (4.8)	72.1 (7.4)
Mean height (m) (SD)	1.66 (0.08)	1.65 (0.06)	1.64 (0.06)	1.64 (0.06)	1.64 (0.06)	1.64 (0.06)	1.62 (0.10)	1.55 (0.08)	1.53 (0.05)	1.52 (0.05)	1.52 (0.05)	1.52 (0.05)	1.52 (0.05)	1.50 (0.08)
Mean body mass index (kg/m^2^) (SD)	17.7 (0.9)	20.1 (0.7)	22.0 (0.6)	24.0 (0.6)	25.9 (0.6)	28.1 (0.8)	31.7 (2.3)	17.6 (0.9)	20.0 (0.7)	22.0 (0.6)	24.0 (0.6)	25.9 (0.6)	28.2 (0.8)	32.1 (2.4)
Weight change since 20 years old (%)														
≤-10.0kg	16.9	8.5	3.8	2.4	1.7	1.5	0.9	14.6	6.7	3.1	0.9	0.8	0.3	0.3
-9.9kg∼-5.0kg	30.0	21.8	13.5	7.0	3.5	2.1	1.5	31.7	22.8	15.3	5.9	2.7	1.3	0.6
-4.9kg∼+4.9kg	49.3	62.4	58.2	37.3	14.0	5.8	4.4	50.8	60.0	53.2	36.5	18.5	6.9	3.2
+5.0kg∼+9.9kg	2.3	6.2	19.2	32.3	30.1	17.8	10.8	2.2	9.5	23.1	36.1	34.8	21.3	9.0
+10.0kg∼+19.9kg	1.5	1.2	5.1	20.4	46.6	55.8	36.3	0.8	1.0	5.3	20.3	40.9	58.1	40.5
+20.0kg≤	0.0	0.0	0.2	0.6	4.1	16.9	46.2	0.0	0.0	0.1	0.3	2.3	12.0	46.3
Education (%)														
≤15	41.5	42.0	40.7	40.0	40.0	40.9	42.9	36.0	35.6	36.5	39.5	43.0	46.5	51.4
16-18	41.2	45.7	45.9	46.1	44.8	44.5	43.2	48.6	49.5	49.9	47.7	46.2	43.7	41.2
19≤	17.4	12.4	13.4	13.9	15.2	14.6	13.8	15.4	15.0	13.6	12.8	10.9	9.8	7.4
Living with spouse (%)														
Yes	91.2	90.6	92.6	92.9	93.9	93.4	91.1	83.7	86.9	87.0	89.5	88.4	87.3	83.3
No	8.8	9.4	7.4	7.1	6.2	6.6	8.9	16.3	13.1	13.0	10.5	11.7	12.7	16.7
Green vegetables (%)														
≤1-2 times/month	11.6	15.5	14.4	14.5	16.3	16.0	19.4	11.6	9.0	8.1	8.1	8.2	9.1	10.5
1-2 times/week	36.8	34.8	34.9	34.9	36.3	35.7	36.9	27.3	31.4	28.7	29.2	30.5	30.2	31.4
3-4 times/week	29.8	29.4	30.0	29.7	28.8	29.4	26.2	35.8	33.7	36.0	36.1	34.6	35.2	35.0
Everyday	21.9	20.4	20.7	20.9	18.6	18.9	17.5	25.3	25.9	27.2	26.7	26.7	25.5	23.2
Oranges (%)														
≤1-2 times/month	32.7	30.5	29.9	28.9	29.7	29.2	29.7	18.5	16.8	15.2	14.8	15.2	17.0	18.8
1-2 times/week	27.4	29.3	30.1	30.0	30.0	29.6	29.7	21.1	20.7	20.4	20.6	20.9	21.5	20.0
3-4 times/week	23.0	23.6	22.7	23.3	21.7	22.7	20.9	25.6	27.7	28.1	26.8	27.0	26.5	25.0
Everyday	17.0	16.6	17.3	17.8	18.6	18.6	19.5	34.8	34.9	36.3	37.9	36.9	35.0	36.3
Smoking status (%)														
Never	13.9	15.0	17.5	20.7	23.5	23.3	22.2	83.2	86.7	89.6	91.7	90.9	90.7	85.3
Past	13.3	14.3	17.4	21.6	23.4	23.8	27.1	3.0	3.0	1.9	1.5	1.4	2.1	3.5
1-19 cigarrets/day	21.2	19.1	16.7	14.6	11.9	11.3	9.2	8.4	7.7	6.3	4.9	5.3	4.7	6.7
20≤ cigarrets/day	51.6	51.5	48.4	43.1	41.1	41.6	41.5	5.5	2.7	2.2	1.9	2.3	2.6	4.5
Alcohol drinking (%)														
Never	21.3	17.1	15.9	15.3	14.5	15.8	18.7	71.3	69.8	69.0	70.1	72.0	71.9	69.0
Past	9.7	6.5	5.7	5.3	5.9	6.2	8.1	5.1	3.7	3.4	3.5	3.1	4.0	7.2
Current	69.1	76.4	78.4	79.4	79.7	78.0	73.2	23.6	26.5	27.6	26.4	25.0	24.1	23.8
Walking (%)														
1 hr/day≤	45.9	47.6	49.0	44.8	41.7	43.4	37.5	45.1	46.0	46.5	46.1	45.1	47.0	37.5
<1 hr/day	54.1	52.4	51.0	55.2	58.3	56.6	62.5	54.9	54.0	53.6	53.9	54.9	53.0	62.5

SD: standard deviation

### Mortality by BMI Categories

Table 2 presents RRs for all-cause mortality according to categories of BMI. Age-adjusted analysis and multivariate analysis with or without the exclusion of subjects who died during the first three years of follow-up showed that there were statistically significant elevations in mortality risk in lean men and women and in obese women. Compared with a referent BMI group (23.0-24.9), multivariate RRs [95% CI] with the exclusion of deaths occurring in the first three years of follow-up were 2.06 (1.49-2.84) and 1.83 (1.17-2.88) for men and women, respectively, in the lowest BMI category (<18.5). The RR (95% CI) was 1.64 (1.09-2.49) for women in the highest BMI category (≥30.0). In contrast, RRs were not significantly increased or decreased among men across the BMI range of 18.5 or higher and among women across the BMI range of 18.5 through 29.9.

**Table 2.** Relative risk (RR) and 95% confidence interval (CI) of all cause mortality by body mass index categories in men and women.^†^

	Body Mass Index						

	<18.5	18.5-20.9	21.0-22.9	23.0-24.9	25.0-26.9	27.0-29.9	30≤
Men							
Person-years	3,603	30,136	51,932	55,414	31,107	17,137	3,770
No.of death	56	217	297	301	147	85	18
Age-adjusted RR	2.50 (1.88 - 3.33)	1.28 (1.07 - 1.52)	1.04 (0.89 - 1.22)	1.00	0.89 (0.73 - 1.08)	0.95 (0.75 - 1.21)	0.94 (0.59 - 1.52)
Multivariate RR1	2.05 (1.53 - 2.74)	1.10 (0.92 - 1.31)	0.97 (0.83 - 1.15)	1.00	0.92 (0.76 - 1.12)	1.01 (0.80 - 1.29)	1.00 (0.62 - 1.60)
Multivariate RR2	2.06 (1.49 - 2.84)	1.12 (0.92 - 1.36)	0.98 (0.82 - 1.17)	1.00	0.96 (0.77 - 1.18)	1.05 (0.81 - 1.36)	0.85 (0.49 - 1.49)

Women							
Person-years	5,711	34,164	54,484	55,463	37,507	23,935	6,865
No.of death	29	75	126	144	95	64	34
Age-adjusted RR	1.96 (1.31 - 2.92)	0.94 (0.71 - 1.24)	0.95 (0.75 - 1.21)	1.00	0.90 (0.70 - 1.17)	0.95 (0.71 - 1.28)	1.72 (1.19 - 2.50)
Multivariate RR1	1.76 (1.16 - 2.67)	0.88 (0.66 - 1.17)	0.92 (0.72 - 1.17)	1.00	0.91 (0.70 - 1.17)	0.95 (0.70 - 1.27)	1.65 (1.13 - 2.40)
Multivariate RR2	1.83 (1.17 - 2.88)	0.82 (0.60 - 1.14)	0.92 (0.70 - 1.19)	1.00	1.02 (0.78 - 1.34)	0.93 (0.67 - 1.28)	1.64 (1.09 - 2.49)

† : Adjusted for age in years; weight loss of 5 kg or more since 20 years old (yes, no); marital status at baseline (whether or not living with spouse; cigarette smoking (never smokers, past smokers, current smokers smoking 1-19 cigarettes per day, or current smokers smoking at least 20 cigarettes per day); alcohol drinking (never drinkers, past drinkers, or current drinkers); walking time per day (less than 1 hour, or 1 hour or longer). Multivariate RR2 has been estimated with the exclusion of 263 subjects (174 men and 89 women) who died within the first 3 years of follow-up. Numbers in parentheses are 95% confidence intervals.

### Effect Modification

We detected statistically significant effect modification of the association between BMI and mortality according to education, consumption frequencies of green vegetables and oranges. The increased risk of all-cause mortality associated with underweight in men and women and overweight in women were more remarkable among subjects with lower education and infrequent consumption of green vegetables and oranges, as compared with subjects with higher education and frequent consumption of green vegetables and oranges (data not shown). We analyzed the association between BMI and mortality after stratifying subjects by smoking status or a weight change since 20 years old, but did not observe substantial effect modification by the two variables (data not shown). For other variables, we also did not detect substantial modification of the association between BMI and mortality (data not shown).

## DISCUSSION

Our prospective cohort study demonstrated that there were statistically significant elevations in mortality risk in obese (BMI≥30.0) women and lean (BMI<18.5) men and women. Subjects with the BMI categories of 18.5 or higher in men and the BMI categories of 18.5 to 29.9 in women had no significant relations between BMI and overall mortality risk in this cohort.

The impact of obesity on mortality remains controversial. Although some results from previous studies have showed that obese subjects had much higher risks of death than those of normal weight subjects,^4^^,^^5^^,^^8^^-^^24^ other results have not showed the elevation of the risks.^4^^,^^10^^,^^18^^,^^19^^,^^21^^,^^24^^-^^29^ This discrepancy have been attributed to failure to properly take cigarette smoking into account and lack of exclusion of individuals with weight loss-related illness.^6^

We adjusted for smoking status and in an attempt to account for the effects of preclinical illness on body weight, we excluded subjects who had history of cancer, stroke, myocardial infarction, kidney disease, or liver disease at baseline. We also conducted analyses that excluded deaths occurring in the first three years of follow-up and that adjusted for a weight loss since 20 years old. Despite these efforts, we did not find an increased risk at the high end of BMI in men. The most plausible explanation for this may be the lack of a sufficient number of men with highest BMI level in our cohort. Only 363 (1.9%) men had a BMI level of 30.0 or over. Therefore, our ability to detect any possible increased risk of mortality in the obese was limited. In contrast, we observed significant elevation of mortality risk in 658 (3.2%) women with highest BMI level, the number of whom was almost doubled to that of men.

We also demonstrated that mortality was high for subjects with lowest BMI (BMI<18.5) both in men and women. In this study, the increased risk of death in these subjects persisted even after the exclusion of early deaths, and adjustment of smoking status and weight change. It is, therefore, not likely that the increased risk from leanness could be fully explained by uncontrolled bias due to preexisting disease processes. Underweight may be an important determinant of premature death among the Japanese population.

We did not observe significant differences in mortality for overweight subjects with BMI of 25.0 to 29.9 both in men and women. Although we could not explain this finding, the result is consistent with that from recent large-scale study of 235,398 men in Korea, in which subjects with BMI of 24 to 29 had risks of death from 0.89 to 0.93 (reference; BMI of 22 to 23), which did not differ significantly from 1.00.

The measure of adiposity that we used in our study has some limitations. First, our measurements of BMI are based on self-reports. Although self-reported weight and height are highly correlated with measured weight and height in the present study, a small, generally systematic, error exists - an overestimation of height and underestimation of weight, especially at heavier weight.^33^ Thus, our measure of BMI probably underestimated the true BMI of overweight subjects, which might make our estimates of the effects of obesity on mortality conservative. Second, we had no measure of central adiposity, such as the ratio of waist circumference to hip circumference. Because greater visceral adiposity is a better predictor of the risk of diabetes or death than the BMI,^34^^,^^35^ measurement of central adiposity would be needed in further studies to estimate the impact of obesity upon mortality.

In conclusion, obese (BMI≥30.0) women and lean (BMI<18.5) men and women had a statistically significant elevations of risks of death, as compared with those with normal weight, in this prospective cohort study of middle-aged rural Japanese population.
